# Cardiovascular Disease Self-Management: Pilot Testing of an mHealth Healthy Eating Program

**DOI:** 10.3390/jpm4010088

**Published:** 2014-03-19

**Authors:** Leila Pfaeffli Dale, Robyn Whittaker, Helen Eyles, Cliona Ni Mhurchu, Kylie Ball, Natasha Smith, Ralph Maddison

**Affiliations:** 1 National Institute for Health Innovation, University of Auckland, Private Bag 92019, Auckland Mail Center, Auckland 1142, New Zealand; E-Mails: r.whittaker@auckland.ac.nz (R.W.); h.eyles@auckland.ac.nz (H.E.); c.nimhurchu@auckland.ac.nz (C.N.M.); nsmi117@aucklanduni.ac.nz (N.S.); r.maddison@auckland.ac.nz (R.M.); 2 Centre for Physical Activity and Nutrition Research, Deakin University, Melbourne Burwood Campus, 221 Burwood Highway, Burwood, VIC 3125, Australia; E-Mail: kylie.ball@deakin.edu.au

**Keywords:** text messaging, health behavior, cardiovascular disease, diet

## Abstract

Cardiac rehabilitation (CR) is crucial in the management of cardiovascular disease (CVD), yet attendance is poor. Mobile technology (mHealth) offers a potential solution to increase reach of CR. This paper presents two development studies to determine mobile phone usage in adults with CVD and to evaluate the acceptability of an mHealth healthy eating CR program. Methods: CR attendees were surveyed to determine mobile phone usage rates. A second single-subject pilot study investigated perceptions of a 4-week theory-based healthy eating mHealth program and explored pre-post changes in self-efficacy. Results: 74 adults with CVD completed the survey (50/74 male; mean age 63 ± 10). Nearly all had mobile phones (70/74; 95%) and used the Internet (69/74; 93%), and most were interested in receiving CR by text message (57/74; 77%). 20 participants took part in the healthy eating pilot study. Participants read all/most of the text messages, and most (19/20) thought using mobile technology was a good way to deliver the program. The website was not widely used as visiting the website was reported to be time consuming. Exploratory *t*-tests revealed an increase in heart healthy eating self-efficacy post program, in particular the environmental self-efficacy subset (Mean = 0.62, SD = 0.74, *p* = 0.001). Conclusions: Text messaging was seen as a simple and acceptable way to deliver nutrition information and behavior change strategies; however, future research is needed to determine the effectiveness of such programs.

## 1. Introduction

A widely supported aspect of cardiovascular disease (CVD) self-management and secondary prevention is cardiac rehabilitation (CR). CR is a hospital or community-based program designed to educate patients about their cardiovascular risk factors and encourage lifestyle change, and has been shown to slow or reverse the progression of CVD and reduce mortality [1,2]. Despite the benefits, rates of participation are low in all countries in which they have been measured [3,4,5,6]. Common barriers to attending and completing CR include lack of time or transport to attend center-based sessions [7,8,9].

Mobile and wireless technologies (or mHealth) offer a viable approach to deliver CVD self-management programs in a way that minimizes disruption to people’s lives. Components of CR can be sent directly to patients’ mobile phones in personalized messages, accessed at any time and any place, thus reducing geographic and time barriers for those who cannot access center-based programs. Such mHealth CR programs could also be implemented to augment existing services by delivering additional long term support to patients. It has been shown to take 6 months of persevering with a new behavior to result in sustained lifestyle change [10] therefore mHealth may be a cost-effective method of delivering longer CR programs.

A common criticism of mHealth is that it creates a digital divide where some may lack access to affordable mobile technologies or the knowledge to operate mobile devices. These concerns may be unfounded as mobile phone saturation has reached 128% in high income countries and 89% in developing countries [11] and Internet use has doubled worldwide in the last five years [12,13,14]. CVD occurs most commonly in middle-to-older age and it is often perceived that older adults are not familiar with mobile technologies. Limited research disputes the digital divide [8,15], however, it is not explicitly known if, or what type of mobile technologies would be a suitable option for this group.

Emerging evidence to date for mobile interventions is promising. Recent systematic reviews have found text messaging and Internet-based interventions effective in achieving behavior change outcomes such as smoking cessation, physical activity, diet, and medication adherence for both disease prevention and management [16,17,18,19]. Such lifestyle modifications are important for controlling many non-communicable diseases, including diabetes and CVD. The recent HEART (Heart Exercise and Remote Technologies) randomized controlled trial (N = 171) found a text messaging and Internet intervention was effective and cost-effective for increasing leisure-time physical activity and walking, but failed to increase maximal oxygen uptake in people with CVD at 6 months [20,21]. The HEART trial focused only on physical activity behavior whereas CR can involve modification of numerous lifestyle factors, including smoking cessation and healthy eating. To address this, a comprehensive mHealth CR intervention is planned, but a first step was to develop and evaluate a healthy eating program.

To date there has been little investigation into the use of mHealth as a tool for healthy eating intervention in adults with CVD. Here we use an established framework [22] to present two development studies, aimed at (1) determining the degree to which people with CVD engage with mobile technology and their interest in this type of intervention; and (2) evaluating the acceptability of an mHealth healthy eating CR program.

## 2. Methods

### 2.1. Overall Design

**Figure 1:**
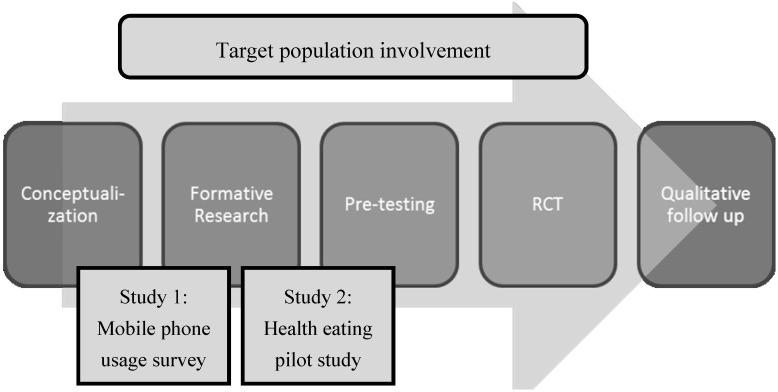
The mHealth Development and Evaluation Framework [22].

The healthy eating CR program was created according to the mHealth Development and Evaluation Framework [22]. The framework follows an iterative process for developing mHealth interventions with end-user engagement. Figure 1 outlines the process used for this paper. The methods and rationale for each step will be described, beginning with conceptualization. Protocols for both development studies received ethics approval (University of Auckland Human Participants Ethics Committee: 8652 and Health and Disability Ethics Committee: NTY/11/07/078) and all participants gave informed consent. The two studies were conducted concurrently in 2012, with two different samples, in Auckland, New Zealand.

### 2.2. Step 1: Conceptualization

A content advisory group comprised of experts in the fields of CR, behavior change, public health, mobile technology, and nutrition met weekly over a 6-month period to develop the healthy eating program. The group was experienced in mHealth interventions [8,20,23]. The healthy eating program consisted of evidence-based information [24] and behavior change strategies aimed at reducing risk of subsequent events and enhancing self-management. Intervention content included healthy eating advice, following the cardioprotective dietary pattern. The guidelines recommend large servings of fruit and vegetables, whole grains, lean meats and fish, and low-fat dairy, and has been shown to reduce cardiovascular and total mortality [24].

The healthy eating behavior strategies were framed in social cognitive theory (SCT) [25]. A key construct of SCT is perceived self-efficacy, which refers to the extent people believe they can exercise control over their health behaviors. Self-efficacy was chosen as a key construct as it has been shown to be both a determinant of health behavior change [25] and a consequence of CR [26]. The healthy eating program aimed to increase self-efficacy by targeting the four sources of influence: mastery experience, vicarious learning, social persuasion, and somatic and emotional states [25]. The program also focused on overcoming barriers to healthy eating, including dining out and giving up favorite foods, which have been identified as significant barriers in the CVD population [26].

### 2.3. Step 2: Formative Research: Mobile Phone Usage among CR Participants across New Zealand

The formative research step investigates initial perceptions of an mHealth program. If the concept is agreeable to the target audience, including participants and key stakeholders, content is developed and then pre-tested by the target audience. In the current study, formative research was conducted to determine the delivery method and the level of interest in an mHealth CR program. Uptake of mobile technology is rapidly increasing across all age groups and it was important to ensure the delivery of the intervention matched participants’ interests and abilities.

Using a cross-sectional design, a convenience sample of adults diagnosed with CVD across New Zealand was recruited via direct contact with existing community based CR education programs. Participants completed a 20-item survey either online, over the phone or on paper at the time of recruitment at local CR sessions (see Supplementary File 1 for survey questions).

### 2.4. Step 3: Health Eating Pilot Study

The pilot study was completed to gain feedback on the usability and acceptability of the healthy eating program. A secondary aim was to explore trends towards changes in self-efficacy to eat a heart healthy diet. A single subject pre-test/post-test design was used and no formal power calculation was considered as this was a pilot study.

Eligible participants were at least 18 years of age, could read and understand English, and had been diagnosed with CVD or self-identified as being at risk of developing CVD due to having medically controlled or high blood cholesterol. Participants were required to have a basic mobile phone capable of receiving text messages, and have access to the Internet. Potential participants were recruited through word of mouth or directly approached at local CR programs. Recruitment continued until the desired number of completed surveys was reached (N = 20). Twenty participants was an attainable, pragmatic target, and this recruitment target was considered sufficient to provide useful feedback and to test the feasibility of the program [27].

Interested participants were emailed a link to the baseline survey, the Heart Healthy Eating Self-efficacy Scale (HHESES) [28], conducted online using LimeSurvey^®^ software [29]. The HHESES is a reliable and valid measure of self-efficacy in people with hypercholesterolemia, a risk factor for developing CVD [28]. It consists of three subscales: heart healthy eating self-efficacy beliefs (Subscale 1), environmental efficacy (Subscale 2), and outcome expectancy (Subscale 3). The first subscale measures one’s confidence to engage in healthy eating habits. Subscale 2 refers to one’s ability to make healthy choices during certain situations, such as eating with friends and family or eating at fast food restaurants. The last subscale measures outcome expectancy, which refers to understanding the benefits of heart healthy eating.

Participants then began receiving one text message per day (28 in total) and had access to the supporting website. Four weeks later participants were contacted by text and email to complete a follow-up online survey, repeating the HHESES and a 32-item feedback questionnaire. Website usage statistics including the frequency, login period, and page views were also tracked.

The healthy eating content was delivered using two mHealth approaches:

1 Text messages: A library of messages was developed providing participants with behavioral support to make healthy dietary changes and increase self-efficacy to change, revolving around a weekly theme (see Table 1 and Supplementary File 2). Mastery experiences, or building successful experiences [25], were created through messages encouraging goal setting and incorporating self-regulation skills to monitor progress to aid in achieving those goals. Social persuasion, or receiving verbal encouragement that one has the skills to succeed [25], was incorporated into the program through encouraging text messages.

**Table 1:** Example text messages.

Theme	Social cognitive theory construct	Message
Lowering my blood cholesterol	Self-regulation	Have you started to look at your nutrition labels? Can you see how much total fat your packaged food contains?
Choosing healthy meats and vegetarian alternatives	Goal setting/Social persuasion	Try replacing red meat with fish. Canned fish counts. See if you can make this change twice this week. You can do it!
Choosing healthy milk and milk products	Mastery experience	Small changes add up—ask the main shopper to switch from butter to a margarine blend. Less cost to your wallet and health!
Packaged foods	Outcome expectation	Think you don’t have the willpower to avoid treat foods or takeaways? Think of your body, your mind, your family.
2 Role model video vignettes and educational Internet support: A library of brief video vignettes was developed to support vicarious learning, as people who observe role model behaviors and their favorable consequences are more likely to remember and repeat the behaviors endorsed by a model [25]. Cardiac patients (role models) were filmed discussing their experiences making dietary change. Brief cooking demonstrations and vignettes from dieticians and health professionals were also offered. Videos were viewed on a secure website where participants could set and review goals, view healthy recipes, meal ideas, and tips, and view links to other relevant web-based resources. The website was programmed to automatically release new content every three to four days, corresponding to the weekly theme.

### 2.5. Analysis

Participation use and satisfaction: Analysis of survey responses and website usage statistics were largely descriptive. IBM SPSS Statistics 20^®^ was used to calculate response percentages. Open-ended responses were coded, compiled into categories, then grouped into themes using a general inductive approach [30]. A summary of results was sent to participants, providing them the opportunity to review and comment on the findings. This served as a member check to improve the credibility and consistency of the results [30].

HHESES: Participants rated their perceived self-efficacy towards healthy eating on a 6-point Likert scale, where 1 = not confident at all, and 6 = completely confident. Scores were calculated by summing across all items in each subscale and dividing by the total number of items in each subscale. Total self-efficacy was scored by averaging the scores of Subscales 1 and 2. Outcome expectancy was calculated by averaging the scores of Subscale 3. Data were extracted from the online survey and imported to IBM SPSS Statistics 20^®^ for analysis. Paired comparisons between the pre and post outcomes were conducted using both parametric (t-test) and non-parametric (Wilcoxon signed rank test) statistics as appropriate. As this was a pilot study all tests were exploratory, and a 5% significant level was used. The small sample size did not allow for further subgroup analysis.

## 3. Results and Discussion

### 3.1. Formative Research: Mobile Phone Usage

Seventy four participants completed the mobile phone usage survey. The majority completed online surveys (59/74), seven were completed on paper and eight surveys were conducted over the phone. The majority of participants were New Zealand European men with a mean age of 63 years (SD = 10) (Table 2). Participants were diagnosed with at least one of the following cardiac events in the previous two years: angina (27/74), myocardial infarction (25/74), atrial fibrillation (8/74), percutaneous coronary intervention (35/74), or coronary artery bypass graft (18/74). Due to the nature of the recruitment strategy, it was not known how many potential participants were approached about the survey; therefore the response rate could not be calculated. The completion rate (the total number of surveys submitted divided by the number of completed surveys) was 86%.

Nearly all participants had a mobile phone (70/74; 26/74 had a Smartphone). Most participants were interested in receiving CR by mobile technology (57/74) and preferred a text message format (53/74), compared with a Smartphone application (13/74) or the Internet (9/74). Ten participants were not interested in mHealth CR because they were not regular mobile phone users. Most participants had access to the Internet (69/74), and used the Internet on a daily basis (54/74). Table 3 illustrates the most commonly used features of mobile phones.

Participants reported they would most like to receive messages on physical activity and healthy eating components of CR. The most useful advice participants learned during CR, summarized in Table 4, included healthy eating and exercise lifestyle changes.

**Table 2:** Participant demographics for the formative research (Study 1) and pilot (Study 2).

Characteristic	Study 1 (*n* = 74)	Study 2 (*n* = 20)
**Gender**		
Male	50	10
Female	24	10
**Age Group** (in years)		
≤40	3	5
41–50	4	4
51–60	19	4
61–70	29	4
71–80	17	3
≥81	2	0
**Ethnicity** ^a,b^		
New Zealand European	50	14
Māori	16	2
Pacific Islander	0	3
Other	7	4

^a^ Totals some participants identified with more than one ethnicity; ^b^ Ethnicity data missing for 1 participant.

**Table 3:** Mobile phone features used.

Feature ^a^	N = 74 (%)
Phone calls	65 (88%)
Text messaging	63 (85%)
Receive videos and/or photos	17 (23%)
Internet search	17 (23%)
Applications	14 (19%)
Instant messaging	5 (7%)
Social networks	6 (8%)

^a^ Participants were able to select all options that apply.

**Table 4:** Most useful cardiac rehabilitation components.

Advice	N (%)
Healthy meal ideas and recipes	47 (64%)
Practical ideas to manage stress	40 (54%)
Setting goals	19 (26%)
Steps to achieve goals	20 (27%)
Exercise ideas	48 (65%)
How to overcome cigarette cravings	1 (1%)
How to remember to take your medications	10 (14%)
Healthy eating tips for takeaways and dining out	33 (45%)

### 3.2. Pilot Testing: Healthy Eating Pilot Study

Twenty people completed the first pilot study survey and were sent the healthy eating program. Ten participants had CVD and were participating in traditional CR and 10 self-identified as being at risk for developing CVD due to having high blood cholesterol. The majority of participants were New Zealand European (14/20) and the mean age was 52 (SD = 15.5) years (see Table 2). Most participants completed the grocery shopping (18/20) and planned/prepared meals (19/20) at least some of the time for their household.

Nearly all participants (19/20) thought using mobile technology was a good way to deliver a healthy eating CR program. All participants reported receiving the text messages and self-reported reading most (10/20) or all (10/20) of the messages. Thirteen participants reported sharing the text messages with family and friends. Participants accessed the website from 0–9 times over the course of the 4 week program (median = 1). Viewing sessions ranged from 1–40 min with a median view time of 4 min. The program was well received. Table 5 displays a descriptive summary of program aspects that participants liked or disliked.

**Table 5:** Nutrition program survey response data (N = 20).

Please rate the following according to whether you liked or disliked them	Liked	Disliked	No comment	Didn’t use
Ideas on how to eat healthier	19	0	1	0
Information on the benefits of healthy eating	18	0	2	0
Information on cooking healthy meals	16	0	3	0
Receiving motivational messages	15	2	1	2
Being supported to feel like I could make these changes	13	1	4	2
Feeling like I belonged/like there were others going through the same thing as me	11	1	6	2
Receiving lots of text messages	10	6	4	0
The website	10	1	3	6
The time of day messages were sent	9	2	9	0
Seeing videos from health professionals	9	0	2	9
Being able to see ‘my goals’ on the website	8	1	3	8
Seeing videos from people like me	4	0	6	10

Themes emerging from open-ended responses are summarized below and are supported with direct written quotes from participants.

1 *Text messaging was a convenient way to deliver healthy eating information*. Participants felt that receiving texts was “quick and easy” and “non-invasive”. The content of the messages was “relevant”, “concise and interesting”.
2 *Texts were encouraging and an effective reminder to make informed healthy food choices*. Participants felt the texts “encouraged and reminded me to make healthy choices”. The texts helped to serve “as alerts of what type of foods are good and are healthy substitutes”.
3 *I’d prefer a more personalized program*. Seven participants commented on how to personalize the program, such as receiving feedback on their progress. Another suggestion was to tailor the time of day the messages were sent out, in order to send a relevant message at a time of day when people often struggled to make the healthy choice, such as “after dinner”. A few participants also mentioned they wanted some personal contact.
4 *Technical and time barriers prevented me from using the website*. Three participants reported problems accessing the website; they forgot their password and revealed it wasn’t a priority to contact the research team for a new password. Some participants also commented that it was too time consuming to view the website, as they were “really busy at work” or “too tired to open the website again at home”.

#### HHESES

Descriptive data for self-efficacy scores are presented in Table 6. Environmental self-efficacy and total self-efficacy scores increased from baseline to follow-up. Scores were higher post-intervention for heart healthy eating self-efficacy and outcome expectancy, but these differences were not statistically significant.

**Table 6:** Descriptive summary of Heart Healthy Eating Self-efficacy scale and subscales.

Scale (Mean ± SD)	Pre-intervention	Post-intervention	Difference (Post–Pre)
Heart healthy eating	4.59 ± 53	4.76 ± 66	0.20 ± 55
Environmental	4.22 ± 71	4.83 ± 70	0.62 ^b^ ± 74
Total self-efficacy ^a^	4.41 ± 59	4.79 ± 66	0.39 ^b^ ± 64
Outcome expectancy	5.22 ± 77	5.37 ± 82	0.15 ± 65

^a^ Total self-efficacy is an average of heart healthy eating and environmental self-efficacy subscales combined; ^b^ Statistically significant difference was detected using both parametric and non-parametric tests (*p* < 0.05).

### 3.3. Discussion

This paper described the results of two studies assessing the usability and acceptability of an mHealth healthy eating program in a CVD population. A key finding from the formative research was that adults diagnosed with CVD used mobile technologies regularly and were interested in receiving CR by mobile phone. These findings speak to the utility of using mobile phones to deliver lifestyle content to this population. While text messaging and the Internet tend to be more popular with younger age groups, media literacy is increasing among adults [31]. Participants preferred a text message format over the Internet, perhaps because text messaging pushes content to passive recipients, whereas accessing a website requires users to actively seek out information. A rate limiting factor for the web-based component in the pilot study was the time it took to log in with passwords, particularly if they were infrequent computer users. For the future trial, step 4 in the framework, the intervention will be delivered primarily by text message with additional information delivered via a more user-friendly website, which will include additional interactive features to promote engagement [32,33].

The pilot study was one of the first to examine the acceptability of an mHealth healthy eating program in a CVD population. Participants found the program useful and acceptable. Participants felt the messages were encouraging and felt supported to make changes to a healthier diet, which reflected the social persuasion source of self-efficacy [25]. Text messages reminded participants to observe what they were eating, which indicated self-regulation concepts were being internalized [33]. Self-efficacy did not appear to be influenced by vicarious learning [25], which was targeted through the video messages on the supporting website, as the majority of participants chose not to comment or did not use the website. Quantitative findings showed an increase in environmental self-efficacy, or confidence to make healthy eating choices when influenced by external factors [28].

Framing the program in SCT was a strength of the pilot study, as theory-based interventions are more likely to be effective [18,19]. Based on the present work and the HEART intervention [20,21], manipulating self-efficacy in an mHealth format may lead to greater behavior change in a CVD population, however other theories and specific behavior change techniques need to be considered [34]. While the changes in self-efficacy were promising, it is important to note that the results should be interpreted with caution as there was no comparison group. The next step is to determine whether changes in self-efficacy translate to healthy eating behavior change.

The pilot study provided important feedback on how to personalize mHealth programs. A review found tailored mHealth interventions were more effective at changing behavior, however few studies had implemented tailored components [19]. Iterations to the healthy eating CR program will include greater tailoring, such as using the participant’s name and delivering messages at the time participants have selected. Bi-directional messaging will be included that allows for personal contact and tailored responses from the study team. This dynamic feedback loop holds promise to improve health behavior as rapid two-way communication provides just-in-time information or strategies to participants [34]. Designing effective automated yet personalized interventions in a cost-effective way is challenging [35], however a personal and multi-faceted approach may enhance motivation to use future programs and lead to improved disease self-management.

A limitation of both studies was the small samples, which were not necessarily representative of the entire CVD population. The technology in the pilot study was also a limitation as participants were required to have access to a mobile phone and the Internet, indicating that enrolled participants were familiar with this technology. Participants were recruited from CR services and non-attenders may have different mobile phone and Internet usage. Future development research should target CR non-attenders as they may benefit most from an mHealth program. Despite the above limitations, the results warrant further investigation into alternative methods for CR delivery.

#### Suggestions for Future Research

Formative research and pilot testing of intervention content have been completed and the next step in the mHealth development and evaluation framework is to conduct a randomized controlled trial. The results from the two development studies in steps 2 and 3, including the iterations described above, will be used to create a comprehensive CR program, aiming to change multiple health behaviors including physical activity, smoking cessation, medication adherence, and healthy eating. Physical activity and smoking cessation components for the comprehensive CR program have already been developed and pre-tested [20,23] and will be refined according to the findings of the healthy eating pilot study. A randomized controlled trial is planned to determine the effectiveness of a comprehensive CR mHealth program to change behavior compared to standard care (control).

## 4. Conclusions

Questions remain over effective mHealth intervention design, including the type and number of behavior change techniques targeted, the appropriate dose of text messages sent, and the type of technology used (text message, video, applications). Development studies lead to better understanding of these issues and therefore more effective trials [22,36]. Reporting how interventions were developed is important as a common limitation of mHealth research is the lack of replicability, as many mHealth interventions vary in their level description [10,36].

The two development studies described above found people with CVD have high usage rates of mobile phones and Internet, and were receptive to a healthy eating mHealth program. Text messaging was seen as a simple and acceptable way to deliver healthy eating information and behavior change strategies and could be integrated as part of a wider mHealth comprehensive CR program.

As the results were from small pilot studies only, further research is needed to determine the effectiveness of such interventions to change behavior.

## Supplementary Files

Supplementary File 1 Supplementary Materials (PDF, 264 KB)

## References

[1] Jolliffe J., Rees K., Taylor R.R.S., Thompson D.R., Oldridge N., Ebrahim S. (2009). Exercise-based rehabilitation for coronary heart disease. Cochrane Database Syst. Rev..

[2] Heran B.S., Chen J.M., Ebrahim S., Moxham T., Oldridge N., Rees K., Thompson D.R., Taylor R.S. (2011). Exercise-based cardiac rehabilitation for coronary heart disease. Cochrane Database Syst. Rev..

[3] Bethell H.J.N., Lewin R.J., Dalal H.M. (2009). Cardiac rehabilitation in the United Kingdom. Heart.

[4] Doolan-Noble F., Broad J., Riddell T., North D. (2004). Cardiac rehabilitation services in New Zealand: Access and utilisation. N. Z. Med. J..

[5] Suaya J.A., Shepard D.S., Normand S.-L.T., Ades P.A., Prottas J., Stason W.B. (2007). Use of cardiac rehabilitation by medicare beneficiaries after myocardial infarction or coronary bypass surgery. Circulation.

[6] Bjarnason-Wehrens B., McGee H., Zwisler A.D., Piepoli M.F., Benzer W., Schmid J.P., Dendale P., Pogosova N.G., Zdrenghea D., Niebauer J. (2010). Cardiac rehabilitation in Europe: Results from the European cardiac rehabilitation inventory survey. Eur. J. Cardiovasc. Prev. Rehabil..

[7] Jones M., Jolly K., Raftery J., Lip G.Y., Greenfield S. (2007). “DNA” may not mean “did not participate”: A qualitative study of reasons for non-adherence at home- and centre-based cardiac rehabilitation. Fam. Pract..

[8] Pfaeffli L., Maddison R., Whittaker R., Stewart R., Kerr A., Jiang Y., Kira G., Carter K., Dalleck L.A. (2012). mHealth cardiac rehabilitation exercise intervention: Findings from content development studies. BMC Cardiovasc. Disord..

[9] Neubeck L., Freedman S.B., Clark A.M., Briffa T., Bauman A., Redfern J. (2012). Participating in cardiac rehabilitation: A systematic review and meta-synthesis of qualitative data. Eur. J. Prev. Cardiol..

[10] Buchholz S.W., Wilbur J., Ingram D., Fogg L. (2013). Physical activity text messaging interventions in adults: A systematic review. Worldviews Evid. Based Nurs..

[11] International Telecommunication Union ICT Facts and Figures. http://www.itu.int/en/ITU-D/Statistics/Documents/facts/ICTFactsFigures2013-e.pdf.

[12] Ofcom Fixed-Line Voice and Mobile Connections Per Head: 2010. http://stakeholders.ofcom.org.uk/market-data-research/market-data/communications-market-reports/cmr11/international/icmr-1.08/.

[13] Commerce Commission New Zealand Annual Telecommunications Monitoring Report 2011. http://www.nbr.co.nz/sites/default/files/images/2011-Annual-Telecommunications-Market-Monitoring-Report-30-April-2012.pdf.

[14] International Telecommunication Union Measuring the Information Society 2011. http://www.itu.int/ITU-D/ict/publications/idi/material/2011/MIS2011-ExceSum-E.pdf.

[15] Parker S.J., Jessel S., Richardson J.E., Reid M.C. (2013). Older adults are mobile too! Identifying the barriers and facilitators to older adults’ use of mHealth for pain management. BMC Geriatr..

[16] Stephens J., Allen J. (2013). Mobile Phone interventions to increase physical activity and reduce weight: A systematic review. J. Cardiovasc. Nurs..

[17] Krishna S., Boren S., Balas E. (2009). Healthcare via cell phones: A systematic review. Telemed. e-Health.

[18] Cole-Lewis H., Kershaw T. (2010). Text messaging as a tool for behavior change in disease prevention and management. Epidemiol. Rev..

[19] Fjeldsoe B.S., Marshall A.L., Miller Y.D. (2009). Behavior change interventions delivered by mobile phone telephone short-message service. Am. J. Prev. Med..

[20] Maddison R., Whittaker R., Stewart R., Kerr A.J., Jiang A., Kira G., Carter K.H., Pfaeffli L. (2011). HEART: Heart exercise and remote technologies: A randomized controlled trial study protocol. BMC Cardiovasc. Disord..

[21] Carter K., Maddison R., Whittaker R., Stewart R., Kerr A., Jiang Y., Pfaeffli L., Rawstorn J. (2013). Heart: Efficacy of a mHealth exercise-based cardiac rehabilitation program. Heart Lung Circ..

[22] Whittaker R., Merry S., Dorey E., Maddison R. (2012). A Development and evaluation process for mHealth interventions: Examples from New Zealand. J. Health Commun..

[23] Whittaker R., Dorey E., Bramley D., Bullen C., Denny S., Elley R., Maddison R., McRobbie H., Parag V., Rodgers A. (2011). A theory-based video messaging mobile phone intervention for smoking cessation: Randomized controlled trial. J. Med. Internet Res..

[24] New Zealand Guidelines Group Evidence-Based Best Practice Guideline: Cardiac Rehabilitation 2002. http://www.health.govt.nz/publication/cardiac-rehabilitation-guideline/.

[25] Bandura A. (1998). Health promotion from the perspective of social cognitive theory. Psychol. Health.

[26] Sharp P.B., Salyer J. (2012). Self-efficacy and barriers to healthy diet in cardiac rehabilitation participants and nonparticipants. J. Cardiovasc. Nurs..

[27] Thabane L., Ma J., Chu R., Cheng J., Ismaila A., Rios L.P., Robson R., Thabane M., Giangregorio L., Goldsmith C.H. (2010). A tutorial on pilot studies: The what, why and how. BMC Med. Res. Methodol..

[28] Gaughan M.E. (2003). Heart healthy eating self-efficacy: An effective tool for managing eating behavior change interventions for hypercholesterolemia. Top. Clin. Nutr..

[29] LimeSurvey Project Team LimeSurvey: An Open Source Survey Tool 2012. http://www.limesurvey.org/.

[30] Thomas D.R. (2006). A general inductive approach for analyzing qualitative evaluation data. Am. J. Eval..

[31] Ofcom Adults Media Use and Attitudes Report: 2012. http://stakeholders.ofcom.org.uk/market-data-research/media-literacy/archive/medlitpub/medlitpubrss/adults-media-use-attitudes/.

[32] Norman G.J., Zabinski M.F., Adams M.A., Rosenberg D.E., Yaroch A.L., Atienza A.A. (2007). A review of eHealth interventions for physical activity and dietary behavior change. Am. J. Prev. Med..

[33] Bandura A. (2004). Health promotion by social cognitive means. Health Educ. Behav..

[34] Riley W., Rivera D., Atienza A., Nilsen W., Allison S., Mermelstein R. (2011). Health behavior models in the age of mobile interventions: Are our theories up to the task?. Transl. Behav. Med..

[35] Klasnja P., Pratt W. (2012). Healthcare in the pocket: Mapping the space of mobile-phone health interventions. J. Biomed. Inform..

[36] Free C., Phillips G., Galli L., Watson L., Felix L., Edwards P., Patel V., Haines A. (2013). The effectiveness of mobile-health technology-based health behaviour change or disease management interventions for health care consumers: A systematic review. PLoS Med..

