# Supplementary material for: Cardiovascular Disease Self-Management: Pilot Testing of an mHealth Healthy Eating Program
Source: J Pers Med. 2014 Mar 19;4(1):88–101. doi: 10.3390/jpm4010088 (PMC4251404; doi:10.3390/jpm4010088)
Supplement: Supplementary File 1 — Supplementary Materials (PDF, 264 KB) [file jpm-04-00088-s001.pdf]

Supplementary File 1: Mobile phone usage survey questions

1.01 Do you have a mobile phone?
O Yes go to 1.02
O No go to 1.03

1.02 If yes, what functions do you use on your mobile phone? (Tick all that apply)
O Phone calls
O Text messaging
O Receive videos and/or photos
O Internet search
O Applications ('apps')
O IM (instant messaging)
O Social networks

1.03 Would you be interested in receiving continuing cardiac rehabilitation support via your mobile phone?
O Yes go to 1.05
O No go to 1.04

1.04 If no, why not?

1.05 If yes, which mobile phone formats would you like? (Tick all that apply)
O Text message
O Video
O Smartphone App
O Internet
O Other:

1.06 If there was a free program that sent text messages to a mobile phone from your cardiac rehabilitation program would you sign up?
O Yes go to 1.07
O No go to 1.08

1.07 If yes, how many messages would you like to receive?
O 2 messages per day
O 1 message per day
O 3-4 messages per week
O 1-2 messages per week

1.08 Which components of cardiac rehabilitation would you most like to receive messages about? (Tick all that apply)
O Taking medications
O Doing regular physical activity
O Eating healthy foods
O Stop smoking
O Stress management
O Other:

1.09 What advice would be most useful to you? (Tick all that apply)
O Healthy meal ideas and recipes
O Practical ideas to manage stress
O Setting goals
O Steps to achieve goals
O Exercise ideas
O How to overcome cigarette cravings
O How to remember to take your medications
O Healthy eating tips for takeaways and dining out
O Other:

1.10 Do you have regular access to the Internet?
O Yes go to 1.11
O No go to 1.12

1.11 If yes, how often do you use the internet?
O everyday
O 3 or more times per week
O once per week
O less than once per week

1.12 Do you have a Smartphone?
O Yes go to 1.13
O No

1.13 If yes, is it an:
O Android phone
O iPhone
O Other Smartphone:

Supplementary File 2. Healthy eating text messages and targeted theoretical constructs.

Weekly theme	SCT construct	Text message
Lowering my blood cholesterol	N/A–Welcome	CRPlus: Thanks for joining the CRPlus study. This is the first of many text messages you will receive to help you with heart healthy eating
Lowering my blood cholesterol	N/A–Study information	CRPlus: Visit the CRPlus website www.crplus.co.nz to watch videos and learn more about cholesterol and healthy eating
Lowering my blood cholesterol	Self-regulation	CRPlus: Have you seen a nutrition information panel on the back of a packaged food? Learn how to read these labels on the website
Lowering my blood cholesterol	Mastery experience	CRPlus: Small changes add up, such as a switch from butter to a margarine blend. Often the same price or cheaper. Less cost to your health too!
Lowering my blood cholesterol	Goal setting	CRPlus: The key to goal setting is to set small goals, commit by writing them down, and revisit them regularly. Try this today on the website
Lowering my blood cholesterol	Vicarious learning Mastery experience	CRPlus: Making the change to a healthy diet can be difficult, but it gets easier if you stick at. Watch the latest video for inspiration
Lowering my blood cholesterol	Outcome expectation	CRPlus: A benefit of eating less saturated fat is lower LDL “lous”' cholesterol. This is good for your arteries and heart
Choosing healthy meats and vegetarian alternatives	Social persuasion–nutrition information	CRPlus: Removing the skin from chicken makes a low-fat heart healthy meal
Choosing healthy meats and vegetarian alternatives	Social persuasion–nutrition information	CRPlus: Fish is lower in saturated fat than beef or chicken. Grill or bake fish for a heart healthy meal
Choosing healthy meats and vegetarian alternatives	Self-regulation	CRPlus: Check the website for tips on meat cooking. Easy changes like cutting off excess fat or skin before cooking reduces saturated fat
Choosing healthy meats and vegetarian alternatives	Goal setting Social persuasion	CRPlus: Try replacing red meat with fish. Canned fish counts. See if you can make this change twice this week. You can do it!
Choosing healthy meats and vegetarian alternatives	Vicarious learning	CRPlus: An easy way to eat less saturated fat: cut fat off meat & choose lean cuts like prime mince. See the latest video and recipe on the website
Choosing healthy meats and vegetarian alternatives	Social persuasion–nutrition information	CRPlus: Lean cuts of meat are lower in fat, but can be more expensive. Save $ by eating smaller portions and fill up with veggies that are in season
Choosing healthy meats and vegetarian alternatives	Social support	CRPlus: Encourage the family to eat healthy with you. Explain why it’s important & ask them to help you set and reach your goals
Choosing healthy milk and milk products	Social persuasion–nutrition information	CRPlus: If you use coconut cream mix with low fat milk or water to reduce saturated fat & coconut cream goes lot further too!
Choosing healthy milk and milk products	Vicarious learning	CRPlus: For more healthy eating tips, watch the latest video and see new recipes on the website
Choosing healthy milk and milk products	Environmental control	CRPlus: Out for a cup of coffee? Try a trim version of your favourite instead,<br>or a long black
Choosing healthy milk and milk products	Goal setting	CRPlus: Set yourself a healthy eating goal each week. Check the website for ideas. This will help you to monitor your progress.
Choosing healthy milk and milk products	Mastery experience	CRPlus: Little steps lead to the best long term progress. Start with one new change per week. Make that your goal!
Choosing healthy milk and milk products	Social support	CRPlus: Dairy doesn't mean unhealthy. Ask the main shopper in your house to choose low fat versions of your favorite yogurt and cheese
Choosing healthy milk and milk products	Mastery experience	CRPlus: It can be hard to make nutrition changes, especially if you don't like the taste. It's easier to make small changes gradually

Supplementary File 2. *Cont.*

Weekly theme	SCT construct	Text message
Packaged foods	Self-regulation	CRPlus: Cut down saturated fat by reaching for a piece of fruit for your snack or dessert today
Packaged foods	Environmental control	CRPlus: Fast food can be part of a healthy diet. Limit yourself to occasional trips and split a meal or order a small size
Packaged foods	Outcome expectation	CRPlus: Think you don't have the willpower to avoid treat foods or takeaways? Think of your body, your mind, and being around for your family
Packaged foods	Social support	CRPlus: Show your family how to check saturated fat on a food label of a favourite snack. Aim for less than 1%, that means 1g/100g serving
Packaged foods	Goal setting	CRPlus: You could save $5/day when you make your own lunch. That's $1200/yr! That's a 7 day trip to Fiji!
Packaged foods	Somatic and emotional states	CRPlus: Too tired after work to cook a healthy dinner? Try getting up a bit earlier and prepare it in the morning or cook on the weekend and freeze
Packaged foods	N/A–Closing message	CRPlus: It's day 28, you have finished the program. Congratulations! Thank you for your participation
Packaged foods	N/A–Closing message	CRPlus: We hope you enjoyed the program. We would appreciate your feedback. Check your email account for the link to the final survey

© 2014 by the authors; licensee MDPI, Basel, Switzerland. This article is an open access article distributed under the terms and conditions of the Creative Commons Attribution license (http://creativecommons.org/licenses/by/3.0/).
